# Dual Optical Signal-based Intraocular Pressure-sensing Principle Using Pressure-sensitive Mechanoluminescent ZnS:Cu/PDMS Soft Composite

**DOI:** 10.1038/s41598-019-51771-z

**Published:** 2019-10-23

**Authors:** Yooil Kim, Sunanda Roy, Gwang-Yong Jung, Jung-Sik Oh, Gi-Woo Kim

**Affiliations:** 1 Department of Mechanical Engineering, Inha University, Incheon, 22212 South Korea; 2 Department of Naval Architecture and Ocean Engineering, Inha University, Incheon, 22212 South Korea

**Keywords:** Mechanical engineering, Sensors and probes

## Abstract

This paper presents a novel principle for intraocular pressure (IOP)-sensing (monitoring) based on a pressure-sensitive soft composite in which a dual optical signal is produced in response to impulsive pressure input. For the initial assessment of the new IOP sensing principle, a human eye is modeled as the spherically shaped shell structure filled with the pressurized fluid, including cornea, sclera, lens and zonular fiber, and a fluid–structure interaction (FSI) analysis was performed to determine the correlation between the internal pressure and deformation (i.e., strain) rate of the spherical shell structure filled with fluid by formulating the finite element model. The FSI analysis results for human eye model are experimentally validated using a proof-of-conceptual experimental model consisting of a pressurized spherical shell structure filled with fluid and a simple air-puff actuation system. In this study, a mechanoluminescent ZnS:Cu- polydimethylsiloxane (PDMS)-based soft composite is fabricated and used to generate the dual optical signal because mechanically driven ZnS:Cu/PDMS soft composite can emit strong luminescence, suitable for soft sensor applications. Similar to the corneal behavior of the human eye, inward and outward deformations occur on the soft composite attached to the spherical shell structure in response to air puffing, resulting in a dual optical signal in the mechnoluminescence (ML) soft composite.

## Introduction

**Figure 1:**
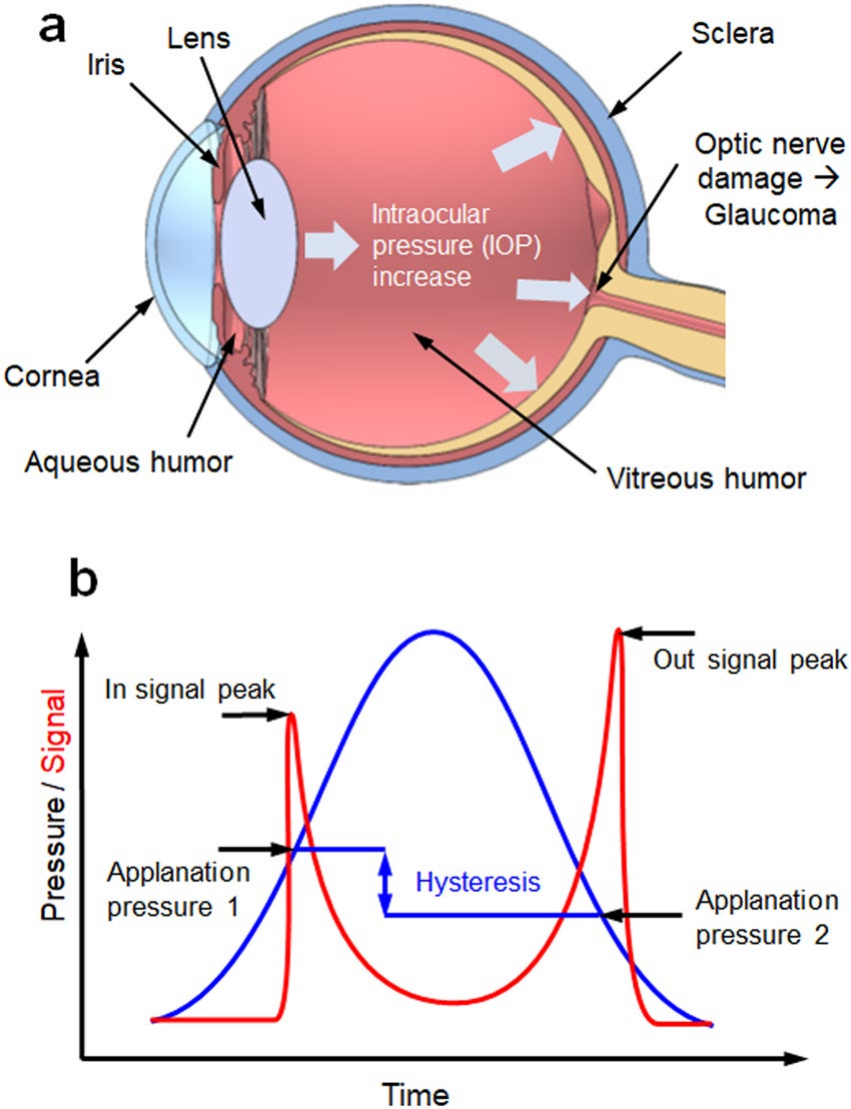
Ocular anatomy of the human eye and state-of-the-art ocular response type tonometry: (**a**) cross-section of the human eye, (**b**) corneal hysteresis-based tonometry. The corneal hysteresis measurement is illustrated on a curve, which compares the corneal applanation signal and the air pressure history over time^14^.

Glaucoma is a chronic eye disease that results in damage to the optic nerve and can lead to vision loss. It is well known that internal eye pressure, commonly referred to as intraocular pressure (IOP), is one of the major risk factors for optic nerve damage. Thus, tonometric measurement of the IOP (i.e., tonometry) is an important diagnostic tool in ophthalmic clinics. IOP is an essential parameter for measuring and preventing the development of glaucoma. As shown in Fig. 1a, the human eye consisting of the cornea, sclera, iris, lens, and zonular fibers is a sensory organ that receives visual information and transmits it to the brain. The eyes have two body cavities; one is the posterior chamber surrounded by the sclera and the lens, and the other is the anterior chamber surrounded by the cornea, the iris, and the lens. Both chambers are filled with aqueous humor. Glaucoma is characterized by optic nerve damage with loss of retinal ganglion cells, and the most important symptom among the various risk factors is an elevated intraocular pressure. This is mainly associated with an increase in the fluid pressure caused by an increase in the aqueous humor in the eyes^1^. Under normal circumstances, the aqueous humor is drained through the trabecular meshwork to maintain an appropriate IOP. However, in glaucoma patients, the trabecular meshwork does not function properly, resulting in an increased IOP. The normal IOP is 10–21 mmHg (mean 15 mmHg ≈ 2 kPa). The difference in binocularity is less than 3 mmHg, and the IOP of a glaucoma patient can be 21 mmHg or more^2^. Typically, glaucoma can be prevented by recognizing and treating an abnormal IOP elevation via periodic IOP sensing. Therefore, an early diagnosis is crucial for preventing glaucoma^3^.

Numerous studies have focused on the measurement of IOP over the past several decades^4,5^. The Goldmann applanation tonometer (GAT) is currently the most widely used tonometer, and it has long been the standard for IOP measurement^6–8^. Applanation tonometry is based on the Imbert–Fick law, which states that the internal pressure of a perfect sphere is equally distributed and the external force required to flatten the sphere is directly proportional to its internal pressure^9^. With the GAT, the required applanation force is measured to estimate IOP based on the Imbert–Fick law. Although the GAT is more accurate than other tonometers, it has disadvantages of requiring anesthesia and difficulty obtaining measurements. Air-puff tonometry (APT) has the advantages of not requiring a topical anesthetic and lacks risk of corneal abrasion, because it uses a noncontact puffing of air to flatten the cornea. As the pressure of the air pulse directed at the cornea increases to deform the cornea, the corneal applanation can be estimated by measuring the reflected pressure. Although APT is a noncontact method, it is inherently inaccurate and needs improvement^10–12^. Unlike prior generations of noncontact tonometers based on the Imbert–Fick law, the ocular response type noncontact tonometer provides the IOP independent of the corneal properties. The light reflected from the central cornea can be collected using a detector (e.g., photo sensors) because the corneal surface behaves like a plane mirror, reflecting light to the detector. The reflected beam will be strongest when the cornea is flat. Then, both the inward and outward applanation can be recorded, as shown in Fig. 1b. The difference between the two values is referred to as the corneal hysteresis, which is proportional to the IOP^13,14^. However, it is difficult to accurately regulate the pressure history of the air puff, which will affect the accuracy of IOP measurement. Thus, although APT is considered a quick and simple method for measuring the IOP of children and other noncompliant patient groups or for high IOP measurements, there is a need for an alternative approach that is more accurate, reliable, and easier to handle.

Therefore, this study primarily aims to develop a new IOP-sensing principle based on a pressure-sensitive ML soft composite from which a dual optical signal is produced. In this study, a mechanoluminescent ZnS:Cu- polydimethylsiloxane (PDMS)-based soft composite is fabricated and used to generate a dual optical signal. Light emission from various organic and inorganic microparticles, such as SrAl_2_O_4_:Eu, Dy (SAO), ZnS:Cu, and ZnS:Mn in response to mechanical stimuli such as friction, force, pressure, and torque is well-known as the mechanoluminescence (ML) phenomenon^15–20^. Recently, there has been a growing interest in the ZnS:Cu/PDMS soft composite because it can emit mechanically driven strong ML and is suitable for flexible soft sensing, light energy harvesting, artificial cochlea, etc.^18–20^. We develop a 3-dimensional (3D) model of the spherical shell structure filled with a fluid that represents the vitreous humor of the human eye. Similar to corneal behavior, the inward and outward deformations occur on the soft composite attached to the spherical shell structure in response to air puffing, resulting in a dual optical signal because of the ML soft composite where the ML is strongly emitted at the maximum strain rates (i.e., inward and outward deformations). This involves a fluid–structure interaction (FSI) analysis that can determine the correlation between the internal pressure and deformation (i. e., strain) rate of the spherical shell structure filled with the fluid. The FSI analysis results are experimentally validated using a proof-of-concept test-bed system consisting of a pressurized spherical shell structure filled with fluid and a simple air-puff actuation system.

## FSI Analysis of Human Eye Model

**Figure 2:**
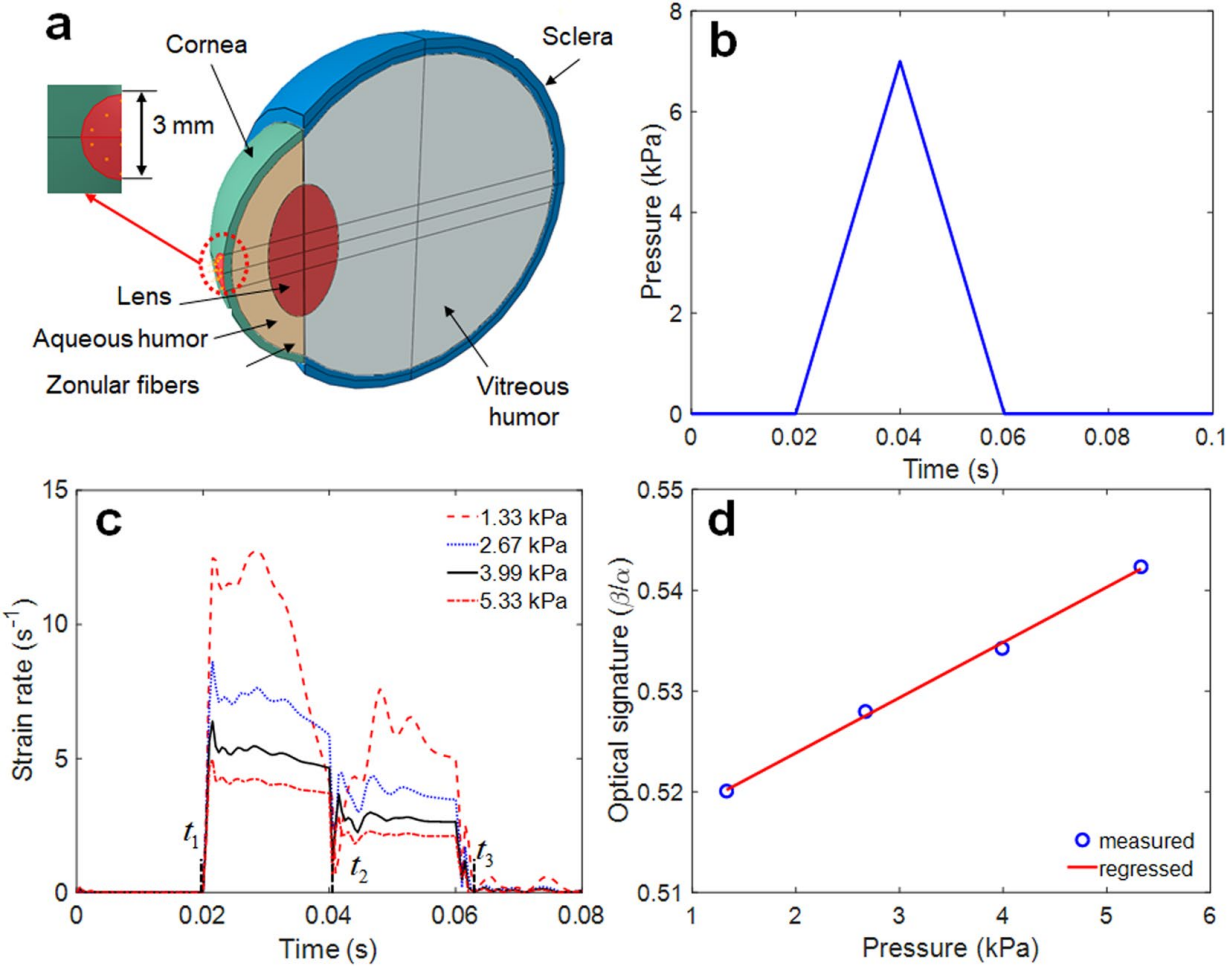
FSI analysis of the human eye model: (**a**) FE mesh model and detailed view of pressure application area; (**b**) applied pressure profile on the center of the cornea; (**c**) strain rate responses; (**d**) sensitivity curve (IOP vs. optical signature).

**Figure 3:**
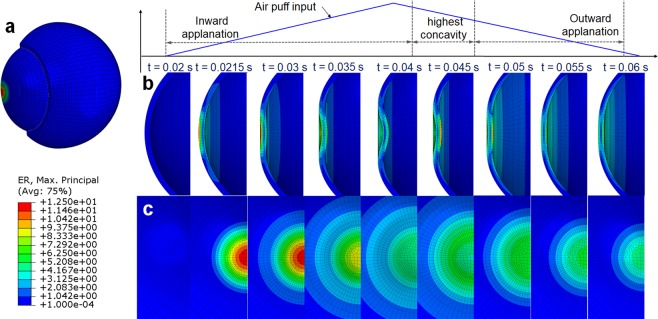
Time evolution of corneal deformation responses to air puff input: (**a**) FE mesh model for FSI analysis (**b**) strain distribution; side view, and (**c**) front view.

For the initial assessment of the new IOP sensing principle, a human eye is modeled as the spherically shaped shell structure filled with the pressurized fluid, including cornea, sclera, lens and zonular fiber. The finite element (FE) model of simple human eye was thus developed to solve a problem of a fluid-coupled structural interaction between shell structure and pressurized fluid, as shown in Fig. 2a. For efficient analysis, the ocular structure of human eye was assumed to be axial symmetry, and the cornea and sclera have a constant thickness and a constant curvature. The iris and ciliary body are neglected, and only zonular fiber is used to analyze. This human eye model was assumed to be filled with a pressurized fluid representing both the vitreous and the aqueous humor. The interaction between solid elements and acoustic elements is defined using a surface-based tie constraint. The thickness and radius of the cornea are 0.55 mm and 8 mm, respectively, and the thickness and radius of the sclera are 1 mm and 11 mm, respectively. The lens was simplified as ellipsoidal solid, and the thickness and radius of the lens are designed to be 4.84 mm and 4.45 mm, respectively. The classical viscoelastic model was then applied for modeling the vitreous humor, where only the first term of the Prony series is used for the time-varying elastic modulus^21^.

1 $$\begin{array}{rcl}{\rm{\sigma }} & = & {\int }_{0}^{t}\,E(t-\tau )d\varepsilon \\ \dot{\sigma }+\frac{1}{\tau }\sigma  & = & {E}_{0}\dot{\varepsilon }+\frac{{E}_{\infty }}{\tau }\varepsilon \\ E(t) & = & {E}_{\infty }+({E}_{0}-{E}_{\infty })\,\exp (-\frac{t}{\tau })\end{array}$$

where *E*_*o*_ is the instantaneous elastic modulus and ${E}_{\infty }$ is the long-time modulus. $\tau$ is the relaxation time, which determines the saturation time for the elastic modulus. The mesh model and the details for the FE model are illustrated in Fig. 2 and Table 1, respectively. The interaction between the spherical shell and the internal pressurized aqueous and vitreous humor, is defined using a surface-based tie constraint. The commercial FE software, ABAQUS, was used for numerical simulation^22,23^. The properties for FE model mesh and parameters for FSI analysis are listed in Table 1 ^24–27^. Implicit nonlinear transient dynamic analysis was then used to calculate the time-varying transient response of a system to impulsive excitation. For the implementation of the noncontact air-puff tonometry, a collimated air pulse with a maximum pressure of 7 kPa and a duration of 40 *ms* was applied as shown in Fig. 2b, and the area incident on the cornea was a circle with a diameter of 3 mm, as shown in Fig. 2a ^28^. FSI analysis was performed at an internal pressure of 1.33 kPa, 2.67 kPa (nearly same as actual normal IOP of 2 kPa), 4 kPa, and 5.33 kPa, respectively. In this study, the strain rate was extracted as a primary design variable of interest because the ML intensity is generally known to be a function of the loading rate^29^. Figure 2c shows the strain rate history at the different internal pressures when the impulsive pressure input (i.e., air puff) is incident on the spherical shell. The dual peaks are observed in the strain rate responses because the strain rate is peaked when the spherical shell was deformed by the incident temporal pressure and again when it was restored by the viscoelasticity of the spherical shell structure. The amplitude of these dual peaks was not equal owing to the hysteresis and their ratio of two peaks seems to be proportional to the internal IOP. The strain rate appeared to transiently vibrate at the 1^st^ and 2^nd^ peak because of the under damped vibration of lens. This dual-peak in strain rate response was similar to the response factor usually used in chromatography. To determine the sensitivity between the dual-peak response and the internal IOP, an optical signature is proposed in this study. The optical signature is defined as the ratio of the area below the intensity curves:

2 $$Optical\,signature\,\frac{\beta }{\alpha }=\frac{{\int }_{{t}_{2}}^{{t}_{3}}\,f(t)dt}{{\int }_{{t}_{1}}^{{t}_{2}}\,f(t)dt}.$$

where *α* and *β* are the area of the first (inward) and second (outward) light emission, respectively, during the entire air puff calculated using numerical integration (trapezoidal method), *f*(*t*) is the strain rate curve, *t*_1_ is the start time of the strain rate, *t*_2_ is the minimum point (i.e., valley) between the first peak and second peak, and *t*_3_ is the end time. The calculated optical signature is linearly proportional to the internal pressure, as shown in Fig. 2d. The sensitivity curve was estimated using the following regressed (curve fitting) formula (*R*^2^ = 0.998):

3 $$y=0.0055x+\mathrm{0.5129.}$$

where *y* denotes the optical signature_1_ and *x* denotes the pressure (kPa). The observation of dual-peak in strain rate response is also confirmed in Fig. 3, which shows the time evolution of corneal deformation responses to air puff input from the FSI analysis. The cornea starts with a convex shape and undergoes three distinct phases: inward applanation (i.e., initial flat deformation); highest concavity; and outward applanation (i.e., recovery). After the inward applanation occurs, the cornea reaches its highest concavity, and convex shape is immediately recovered to its original shape. However, the time evolution of corneal deformation responses is asymmetric about 0.04 s because of the viscoelastic properties of the corneal elements.

**Table 1:** Parameters for FE model and isotropic material properties of human eye.

Component	Young’s Modulus (MPa)	Poisson’s Ratio	Density (kg/m^3^)	No. of Nodes	No. of Elements	Element type
Cornea^24^	0.2	0.495	1100	2640	1672	Solid (Lagrangian element)
Sclera^24^	0.4	0.495	1100	6003	4004	Solid (Lagrangian element)
Lens	0.1	0.47^24^	1000	5125	4438	Solid (Lagrangian element)
Zonular fibers^24^	0.35	0.47	1100	405	176	Solid (Lagrangian element)
Vitreous humor	2.98e-5	0.495^27^	950^25^	23985	20934	Solid (Lagrangian element)
	Relaxation time (s)	1.43^26^	Initial shear modulus (Pa)	10^25^	Infinite shear modulus (Pa)	0.3^25^

## Experimental Validation

**Figure 4:**
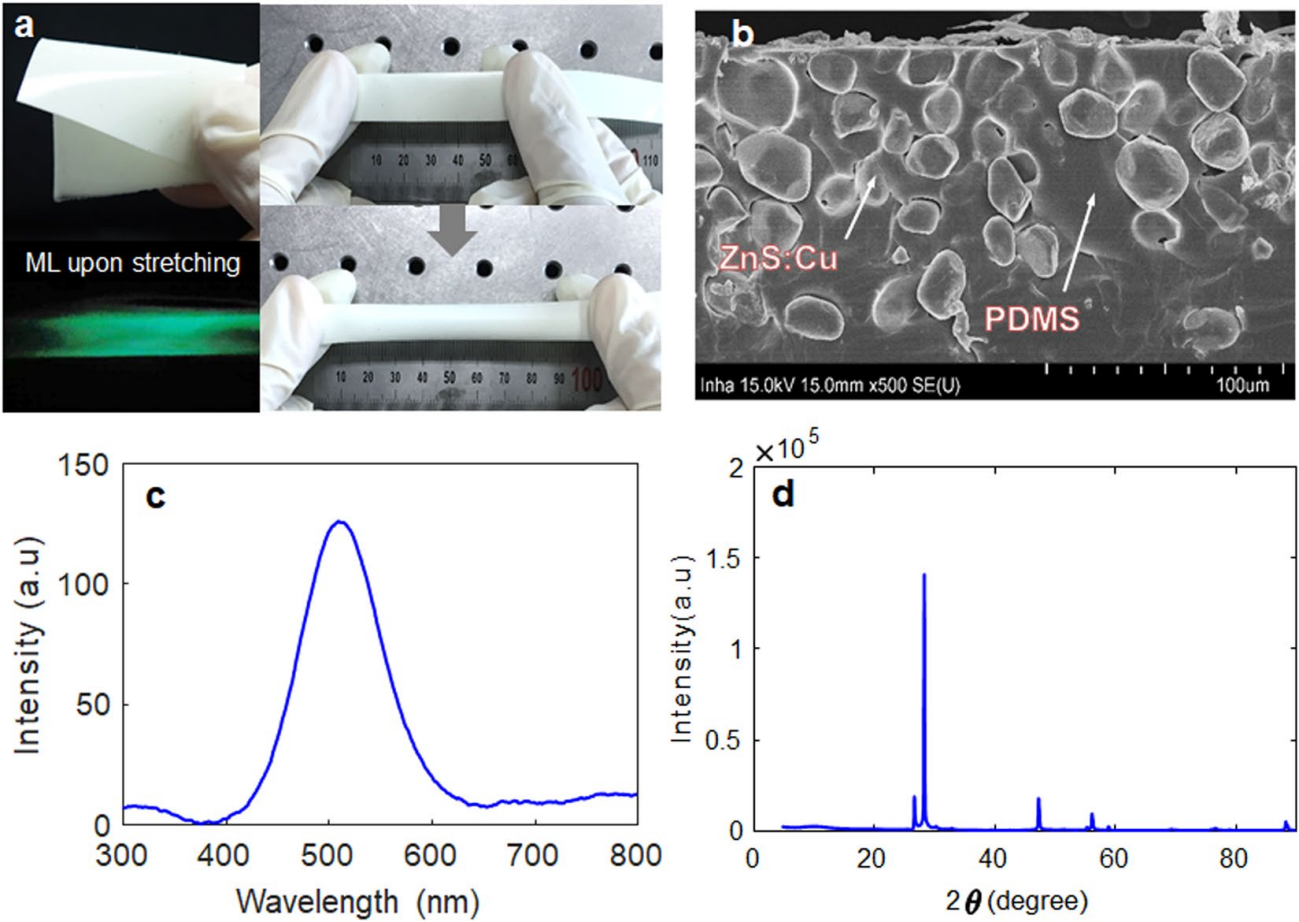
ZnS:Cu-PDMS soft composite (*t* = 0.2 mm, 30 wt.% of ML powder): (**a**) photograph of the ZnS:Cu-PDMS ML elastomeric composite. Inset shows the stretchability of the film and its mechanoluminescence response (green light) when stretched by hand in the dark; (**b**) cross-sectional SEM image showing the embedment and dispersion of ZnS:Cu particles in the PDMS matrix; (**c**) ML spectra; and (d) XRD curve.

Based on the observations from FSI analysis of human eye model, it is possible to provide a new indirect IOP sensing method provided that the dual peaks can be captured by non-contacting optical sensory materials. In this section, ML microparticles are used for optical sensory materials because the ML is typically known to strongly emit at maximum strain rates (i. e, the inward and outward deformations in the human eye model). To induce the dual optical signal from the two applanations (inward and outward), the pressure-sensitive ML soft composite (thickness = 0.2 mm) consisting of 30 wt.% ZnS:Cu microparticles (i.e., powder) is prepared. This specimen was selected for IOP experiment from the four specimens by trading off the flexibility and ML intensity (see supplementary information). This ML soft composite is flexible and highly stretchable (see the top right inset in Fig. 4a). Upon stretching by hand, the film showed excellent ML green visible light in the completely dark environment (e.g. at night), as shown in the bottom inset of Fig. 4a. As confirmed from the microscopic SEM image shown in Fig. 4b, the ZnS:Cu microparticle was uniformly mixed with the PDMS matrix. The X-ray diffraction (XRD) pattern, shown in Fig. 4d, shows the typical peaks of ZnS:Cu nanocrystals, comprising of (111), (220), and (311) lattice planes of a cubic zinc blended structure and are in good agreement^29,30^.

**Figure 5:**
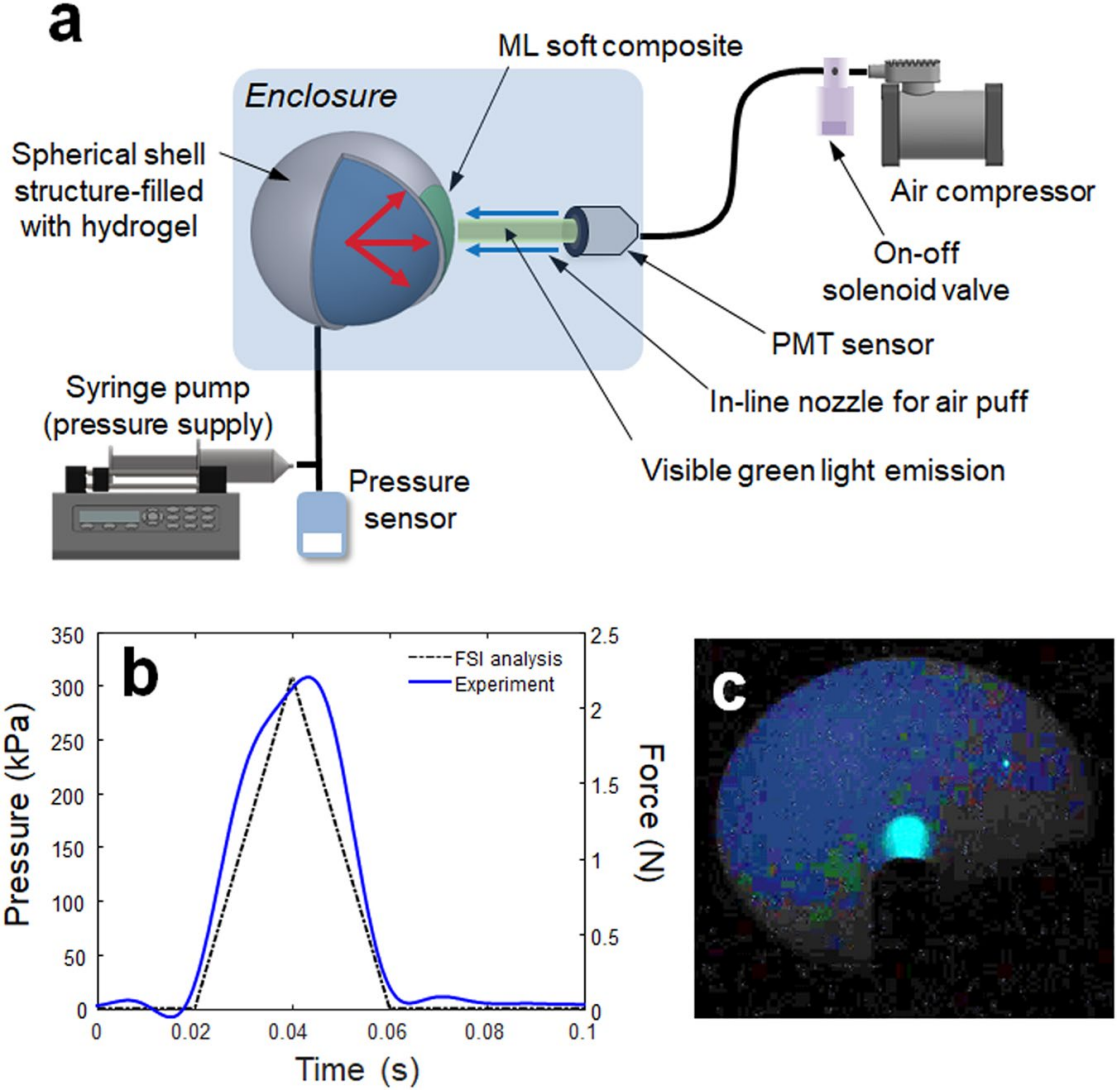
Experimental setup for sensing the internal pressure of the spherical shell structure-filled with hydrogel: (**a**) schematic of overall setup and illustration of light emission; (**b**) temporal pressure and force profile applied at the center of the FSI model and spherical shell used in the experiment^26^; (**c**) accumulated ML image captured by the high-speed camera module (exposure time 30 s).

**Figure 6:**
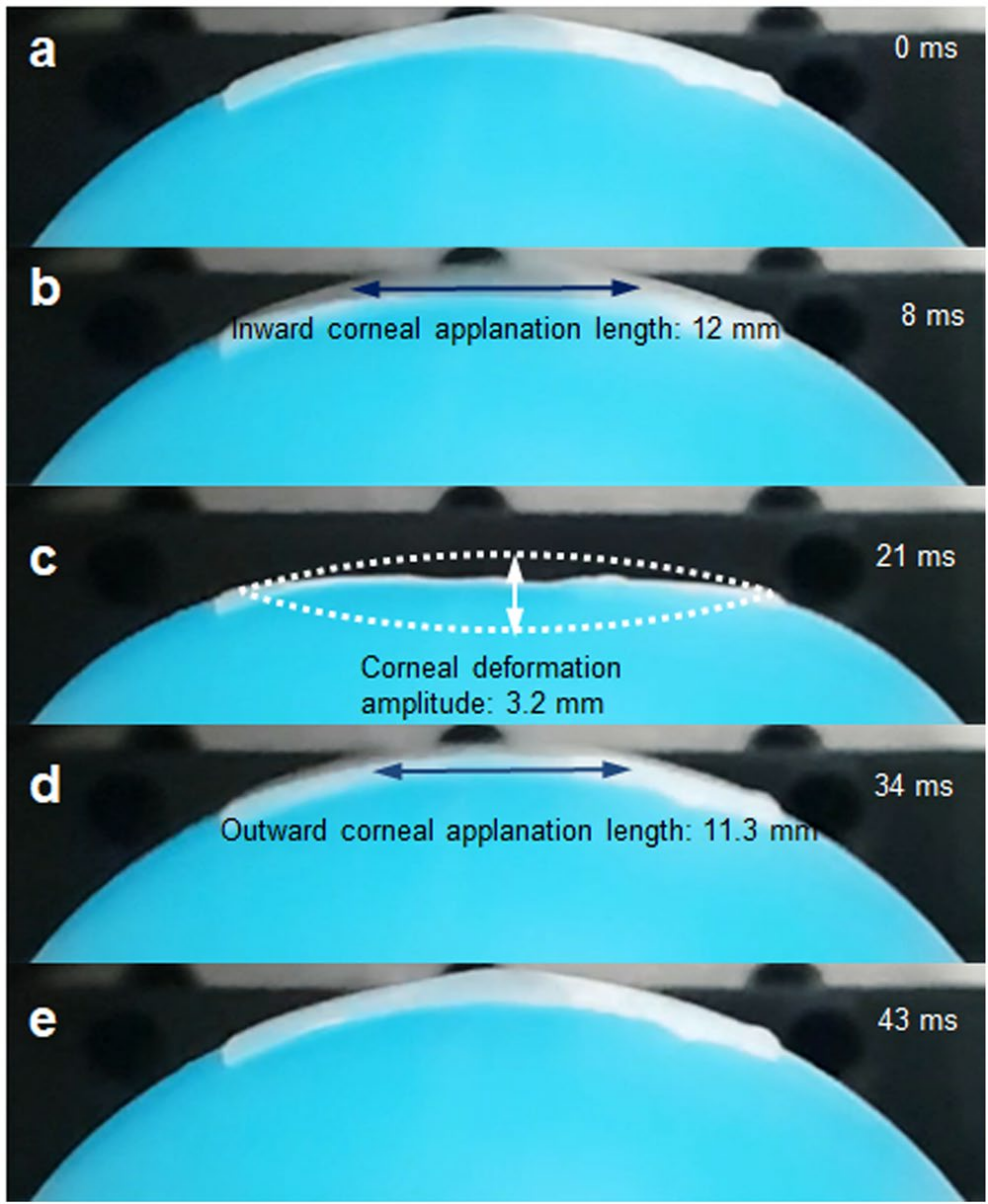
Corneal responses to an air puff captured by high-speed camera: (**a**) before deformation; (**b**) inward applanation; (**c**) highest concavity; (**d**) outward applanation; (**e**) after deformation.

**Figure 7:**
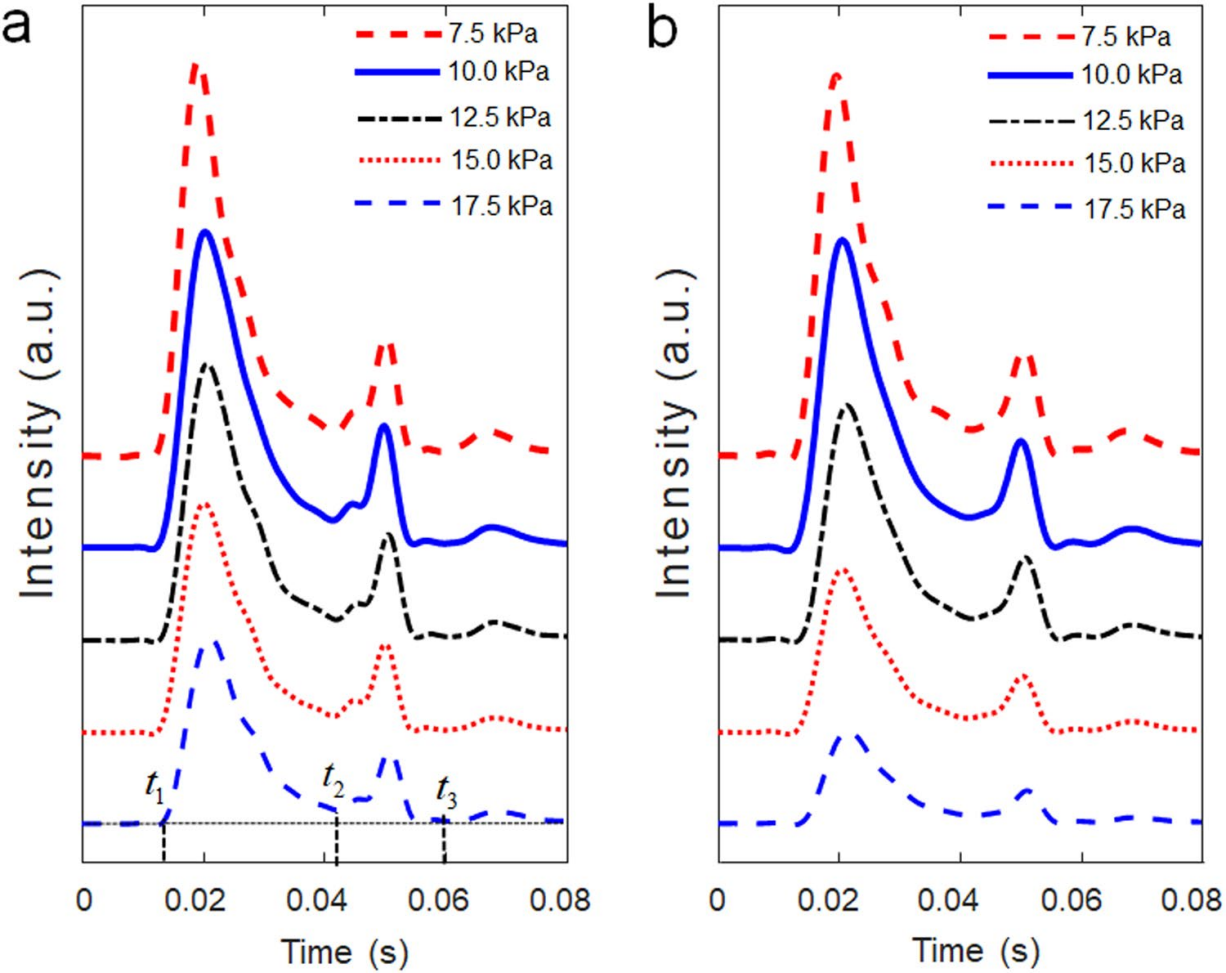
Intensity of ML emission (arbitrary unit, a.u.) at different internal pressures (7.5, 10.0, 12.5, 15.0, and 17.5 kPa) and for the different ML soft composite: (**a**) thickness of 0.2 mm; and (**b**) thickness of 0.6 mm.

**Figure 8:**
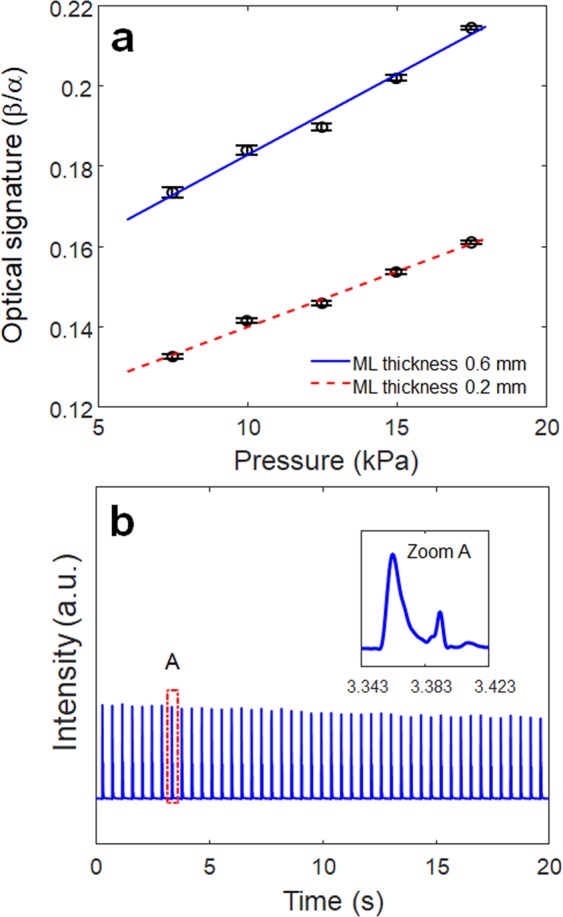
Sensitivity curves for different thicknesses and repeatability of ML soft composite: (**a**) blue bars and blue circles indicate the standard error and average optical signatures (*β*/*α*), the measurements at each internal pressure are repeated 10 times, and the ‘I’ shapes represent the error bars indicating the standard error, and the circular markers represent the average values at each internal pressure; and (**b**) ML intensity history from the ML soft composite (thickness: 0.2 mm) during 45 consecutive cycles.

The effectiveness of the new dual optical signal-based IOP sensing principle was experimentally validated through a laboratory level test-bed system, as shown in Fig. 5a. The human eye model for FSI analysis was further simplified by removing the lens and zonular fiber. The thickness of the spherical shell structure made of a flexible polymer is 0.17 mm, and the ML soft composite (0.2 mm thickness) was attached to the surface of the spherical shell. The inside of the spherical shell was filled with a dilute hydrogel (98% water, viscosity 8.83 Pa·s) to mimic the vitreous humor. It was pressurized using a syringe pump (New Era NE-300). The pressure ranges from 7.5 to 17.5 kPa with increments of 2.5 kPa; this was achieved by an orifice type miniature pressure sensor (Elveflow MPS4L). The spherical shell was excited using an impulsive air pulse with a duration of 40 ms and an amplitude of 2.2 N, as shown in Fig. 5b. The air pulse was controlled by a solenoid control valve (ARO P251SS-012-D) and a relay, and was discharged through a nozzle with an outlet diameter of 3 mm printed with a fused deposition modeling (FDM) 3D printer. To visualize and confirm the ML, a high-speed camera (Sony RX10) was also used to capture the accumulated ML images by setting the exposure time to 30 s, as shown in Fig. 5c. The working principle can be described as follows. The visible-light emission, induced by the deformation of ML on the spherical shell, was measured directly using an in-line photomultiplier tube (PMT) sensor (Hamamatsu H10722). The ML intensity was measured inside an enclosure (i.e., dark environment) for sensitive optical recording and blocking of background light. In real APT, this dark environment can be produced when the eyes of the patients tightly close to block the light. Because the ML intensity emitted from the ML soft composite is typically proportional to the strain rate^29^, the PMT sensor can detect the inward and outward applanation where the maximum strain rate occurs. The high-speed camera was also used to record the dynamic corneal deformation response to the air puff, as shown in Fig. 6. The cornea, with a convex shape, undergoes three distinct phases: inward applanation (i.e., deformation); highest concavity; and outward applanation (i.e., recovery). After the inward applanation is achieved by the air puff, the cornea reaches its highest concavity with a depth amplitude of 3.2 mm, as shown in Fig. 6c. The outward applanation is achieved when the cornea rebounds to its original shape. This dynamic corneal deformation response can also be monitored by measuring the relative intensity in arbitrary units (arb. units) of the light emitted from the ML soft composite using the PMT sensor, as shown in Fig. 7. Similar to the FSI analysis (Fig. 2), the maximum strain rate appears twice, and the ML emission peaks are accordingly observed twice (inward and outward applanation) during the air puff. The optical signature can be estimated by replacing the strain rate curve with ML intensity curve in Eq. (2). The optical signature is linearly proportional to the internal pressure, as shown in Fig. 8a. For a thickness of 0.2 mm, the sensitivity curve is estimated using the following regressed (curve fitting) formula (*R*^2^ = 0.963):

4 $$y=0.0028x+\mathrm{0.11.}$$

where *y* denotes the optical signature_1_ and *x* denotes the pressure (kPa). When the thickness is increased from 0.2 mm to 0.6 mm (thick), the sensitivity curve was estimated by the following regressed formula (*R*^2^ = 0.947):

5 $$y=0.0040x+\mathrm{0.15.}$$

The optical signatures were successfully calculated from the dual optical signals, and exhibits the internal pressure-dependent linear sensitivity. As the thickness of the ML soft composite increased, the stiffness of the ML soft composite increased and the ML emission during the first applanation (deformation) decreased. As a result, the value of the optical signature increased as the thickness of the ML soft composite increased. Meanwhile, the sensitivity curve exhibited no significant change when the thickness of the ML soft composite changed. The repeatability of the internal pressure measurement was also roughly examined using the cycling test. The amplitudes of the signals from the PMT sensor appear to be nearly constant over 45 consecutive cycles (internal pressure in not regulated), as shown in Fig. 8b. Compared to conventional methods, this new principle has some advantageous. For example, it is not necessary to interrogate the reference applanation pressure (the blue solid line in Fig. 1b), whereas the conventional method has to interrogate the reference applanation pressure with pressure sensors to calculate the corneal hysteresis.

## Conclusions

This study demonstrated a proof-of-concept of a new IOP sensing (monitoring) principle based on dual optical signals using a pressure-sensitive ZnS:Cu/PDMS soft composite. The optical signatures were successfully calculated from the dual optical signals measured by PMT sensors, and exhibited the internal pressure-dependent linear sensitivity. For instance, when the internal pressure was 7.5 kPa (actual normal IOP is 2 kPa) and the thickness of the ML soft composite was 0.6 mm, the optical signature calculated from the dual optical signal was approximately 0.175, where the dual optical signal was the most distinct. As the internal pressure increased to 15 kPa, the optical signature also increased to 0.21 (20% change). Based on the FSI analysis and experimental validation, the new method can contribute to current tonometry methods for IOP measurement. However, there remain some technical issues to be further studied. We will expand the proof-of-concept experiment to an actual *in vivo* experiment with a bovine eye. The future direction of ongoing research includes the improvement of sensitivity using a more pressure-sensitive ML soft composite and securing its medical safety.

## Methods

### Fabrication of ML Soft Composite

The ML soft composite was fabricated using commercially available ZnS:Cu (Lonco Company Limited) microparticle powder and PDMS (Sylgard 184 silicone elastomer). First, the ZnS:Cu powder was dispersed in 50 ml of *N*-methylpyrrolidine (97%, NMP) solvent and the mixture was treated with a ultra-probe sonic processor for 30 min to destroy the intrinsic aggregation potential. Then, the ML powder was vacuum filtered, collected, and dried in an oven for 24 h. Approximate amounts of the ML powder (30 and 40 wt.%) were added to a mixture of the liquid prepolymer and the curing agent (10:1), which was mixed thoroughly to form a homogeneous dispersion. The composite mixture was then cast onto a glass Petri dish. Finally, the composites were dried in an oven at 80 °C for 6 h to complete the curing/polymerization process. The solidified thin ML composite (thickness 0.7–0.8 mm) was then peeled off the glass plate and stored for subsequent experiments and characterization.

## Supplementary information

Supplementary Information

Demo movie
