# Supplementary material for: Dual Optical Signal-based Intraocular Pressure-sensing Principle Using Pressure-sensitive Mechanoluminescent ZnS:Cu/PDMS Soft Composite
Source: Sci Rep. 2019 Oct 23;9:15215. doi: 10.1038/s41598-019-51771-z (PMC6811554; doi:10.1038/s41598-019-51771-z)
Supplement: Supplementary file 1 — Supplementary Information [file 41598_2019_51771_MOESM1_ESM.pdf]

Supplementary Information

Dual Optical Signal-based Intraocular Pressure-sensing Principle Using Pressure-sensitive Mechanoluminescent ZnS:Cu/PDMS Soft Composite

Yooil Kim[2], Sunanda Roy[3], Gwang-Yong Jung[1], Jung-Sik Oh[2], and Gi-Woo Kim[1*]

[1] *Department of Mechanical Engineering, Inha University, 22212, South Korea*

[2] *Department of Naval Architecture and Ocean Engineering, Inha University, 22212, South Korea*

*Address correspondence to Gi-Woo Kim, Department of Mechanical Engineering, Inha University, 100 Inha-ro, Nam-gu, Incheon 22212, Republic of Korea. Tel: +82-32-860-7313; Fax: +82-32-868-1716; E-mail: gwkim@inha.ac.kr

Abstract

This paper presents a novel principle for intraocular pressure (IOP)-sensing (monitoring) based on a pressure-sensitive soft composite in which a dual optical signal is produced in response to impulsive pressure input. For the initial assessment of the new IOP sensing principle, a human eye is modeled as the spherically shaped shell structure filled with the pressurized fluid, including cornea, sclera, lens and zonular fiber, and a fluid–structure interaction (FSI) analysis was performed to determine the correlation between the internal pressure and deformation (i.e., strain) rate of the spherical shell structure filled with fluid by formulating the finite element model. The FSI analysis results for human eye model are experimentally validated using a proof-of-conceptual experimental model consisting of a pressurized spherical shell structure filled with fluid and a simple air-puff actuation system. In this study, a

mechanoluminescent ZnS:Cu- polydimethylsiloxane (PDMS)-based soft composite is fabricated and used to generate the dual optical signal because mechanically driven ZnS:Cu/PDMS soft composite can emit strong luminescence, suitable for soft sensor applications. Similar to the corneal behavior of the human eye, inward and outward deformations occur on the soft composite attached to the spherical shell structure in response to air puffing, resulting in a dual optical signal in the mechnoluminescence (ML) soft composite.

Keywords: Mechanoluminescence, ZnS:Cu/PDMS Soft Composite, Intraocular Pressure Sensing, Fluid–Structure Interaction Analysis, Dual Optical Signal

Supplementary #1: FSI Analysis of Experimental Model

To support FSI analysis of human eye model and evaluate the experimental results, a human eye model is further simplified based on the simple human eye model described in Figure 2a. The lens and zonular fiber was removed and cornea is assumed to be smoothly connected by sclera, as shown in Figure S1. In addition, the spherical shell is filled with a pressurized hydrogel representing the viscoelastic vitreous humor of the human eye. The external diameter and thickness of the spherical shell and 65 mm and 0.2 mm, respectively. The mesh model and the isotropic material properties of each element are summarized in Figure S1a and Table S1, respectively. The dual peaks are observed in the strain rate responses (Figure S1c) because the strain rate is peaked when the spherical shell is deformed by the incident temporal pressure and when it is restored by the viscoelasticity of the spherical shell structure. The amplitude of these dual peaks is not equal because of the hysteresis and their ratio of two peaks seems to be proportional to the internal IOP.

Table S1. Details of FE model and isotropic material properties of experimental model

Component	Young's Modulus (MPa)	Poisson's Ratio	Density (kg/m^3)	No. of Nodes	No. of Elements	Element type
Spherical shell	4.8 [2]	0.45 [2]	1200 [2]	5379	3438	Solid (Lagrangian element)
Vitreous humor (hydrogel)	9.3e-5 [calculated]	0.48 [1]	950 [measured]	27201	25230	Solid (Lagrangian element)
	Relaxation time (s)	1.42 [1]	Initial shear modulus (Pa)	31.1 [1]	Infinite shear modulus (Pa)	19.6 [1]

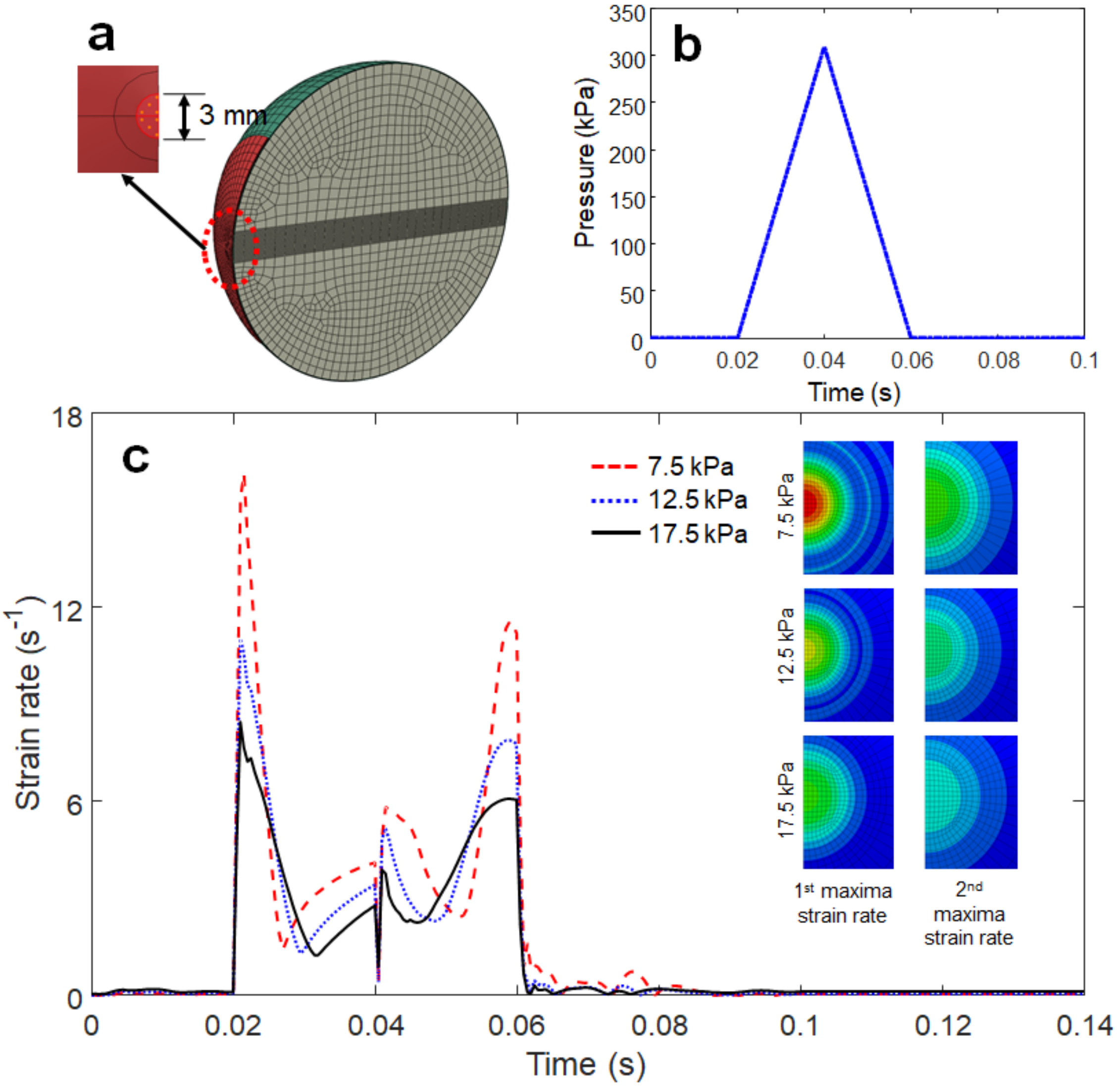

Figure S1. FE model for the spherical shell used in the experimental mesh model (shell thickness: 0.2 mm): (a) spherical shell and hydrogel mesh model and detailed view of pressure application area; (b) applied pressure profile. (c) strain rate responses (inset: contour images for maxima strain rate (front view)) for different internal pressures (7.5, 12.5, and 17.5 kPa).

Supplementary #2: FSI Analysis of Experimental Model with Different Thickness

When the thickness of shell structure increases to 2 mm (close to human eye model), the strain rate histories at the different internal pressures (7, 15, and 23 kPa) are shown in Figure S2c. Similar to the Figure S1, the dual peaks are observed in the strain rate responses, and the amplitude of these dual peaks is not equal and their ratio of two peaks seems to be proportional to the internal IOP. Compare to the thickness of 0.2 mm (Figure S1), the strain rate increases about 1.2 times. In addition, the curves in the middle (0.04 s) is more smooth and similar to human eye model.

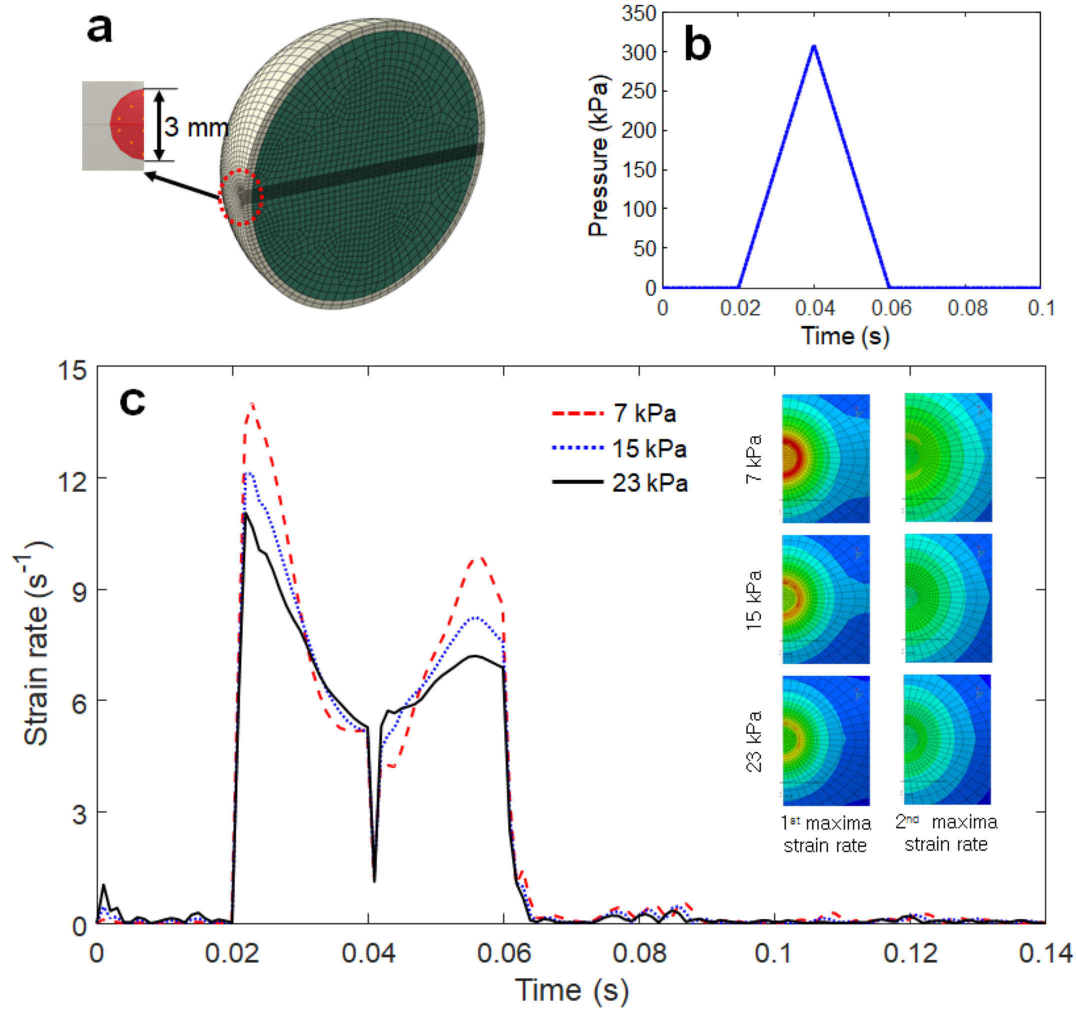

Figure S2. FSI analysis of experimental model (corneal shell thickness: 2 mm): (a) spherical shell and hydrogel mesh model and detailed view of pressure application area; (b) applied pressure profile. (c) strain rate responses (inset: contour images for maxima strain rate (front view)) for different internal pressures (7, 15, and 23 kPa).

Supplementary #3: Methods: Preparation of ML ZnS:Cu/PDMS Soft Composite

In this study, the four specimen of ML soft composite were prepared, and the detail parameters of ML soft composites were listed in Figure S3a. The thickness was changed from 0.2 to 0.6 mm, and weighted percentages (wt.) of ML powder are 30% and 40% are prepared. The intensity of ML soft composite is shown in Figure S3b. When the thickness was increased, the intensity of ML soft composite was increased, and the specimen #2 showed the largest intensity. The elastic mechanical property was also evaluated by tensile test, as shown in Figure S3c. The elastic modulus (i.e., slop in the linear region) of specimen#4 was the lowest but exhibited the most toughness (soft & stretchable). Among the five specimens, the specimen #3 was therefore selected for IOP experiment by trading off the flexibility and ML intensity. As shown in the Figure S3d, the XRD patterns showed the roughly same peaks of ZnS:Cu nanocrystals.

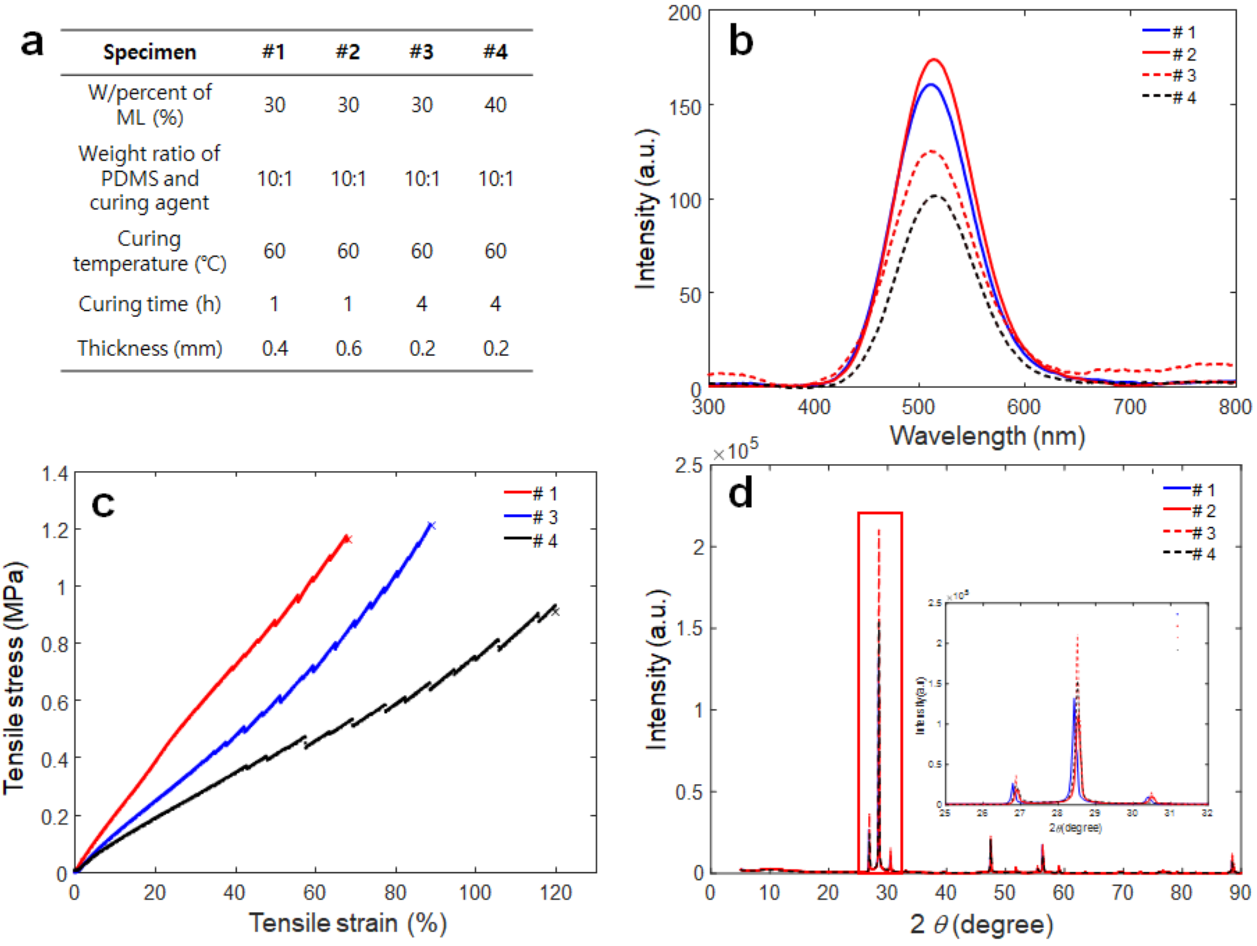

Specimen	#1	#2	#3	#4
W/percent of ML (%)	30	30	30	40
Weight ratio of PDMS and curing agent	10:1	10:1	10:1	10:1
Curing temperature (℃)	60	60	60	60
Curing time (h)	1	1	4	4
Thickness (mm)	0.4	0.6	0.2	0.2

Figure S3. ZnS:Cu/PDMS soft composite: **(**a) specimen (b) ML spectra; (c) tensile test, and (d) XRD curve

References

1. Liu, X. *et al*. Prediction of globe rupture caused by primary blast: a finite element analysis. *Comput. Methods Biomech. Biomed. Engin.* **18**, 1024-1029 (2015).
2. Lee, B., Litt, M. & Buchsbaum, G. Rheology of the vitreous body. Part I: viscoelasticity of human vitreous. *Biorheology* **29**, 521-533 (1992).
